# Penetrating cations induce pleiotropic drug resistance in yeast

**DOI:** 10.1038/s41598-018-26435-z

**Published:** 2018-05-25

**Authors:** Kseniia V. Galkina, Elizaveta G. Besedina, Roman A. Zinovkin, Fedor F. Severin, Dmitry A. Knorre

**Affiliations:** 1 Faculty of Bioengineering and Bioinformatics, Moscow State University, Leninskiye Gory 1–73, Moscow, 119991 Russia; 2 Belozersky Institute of Physico-Chemical Biology, Moscow State University, Leninskiye Gory 1–40, Moscow, 119991 Russia; 3 Institute of Mitoengineering, Moscow State University, Leninskiye Gory 1, Moscow, 119991 Russia; 4 Institute of Molecular Medicine, Sechenov First Moscow State Medical University, Moscow, 119991 Russia

## Abstract

Substrates of pleiotropic drug resistance (PDR) transporters can induce the expression of corresponding transporter genes by binding to their transcription factors. Penetrating cations are substrates of PDR transporters and theoretically may also activate the expression of transporter genes. However, the accumulation of penetrating cations inside mitochondria may prevent the sensing of these molecules. Thus, whether penetrating cations induce PDR is unclear. Using *Saccharomyces cerevisiae* as a model, we studied the effects of penetrating cations on the activation of PDR. We found that the lipophilic cation dodecyltriphenylphosphonium (C_12_TPP) induced the expression of the plasma membrane PDR transporter genes *PDR5*, *SNQ2* and *YOR1*. Moreover, a 1-hour incubation with C_12_TPP increased the concentration of Pdr5p and Snq2p and prevented the accumulation of the PDR transporter substrate Nile red. The transcription factor *PDR1* was required to mediate these effects, while *PDR3* was dispensable. The deletion of the *YAP1* or *RTG2* genes encoding components of the mitochondria-to-nucleus signalling pathway did not prevent the C_12_TPP-induced increase in Pdr5-GFP. Taken together, our data suggest (i) that the sequestration of lipophilic cations inside mitochondria does not significantly inhibit sensing by PDR activators and (ii) that the activation mechanisms do not require mitochondria as a signalling module.

## Introduction

Living cells are capable of resisting toxic compounds due to the activity of plasma membrane pumps with broad substrate specificity. These pumps extrude various chemically unrelated molecules from the cytoplasm to the media (see reviews)^1,2^. Such a protection requires an energy investment because such pumps consume ATP. Accordingly, *Saccharomyces cerevisiae* cells with inactivated pleiotropic drug resistance (PDR) ABC transporters show increased ATP concentrations and reach stationary phase cell densities that are higher than those of the wild-type cells^3^. Moreover, under normal conditions, the overexpression of a major pleiotropic drug resistance ABC transporter *PDR5* decreases the fitness of the strain^4^. For this reason, cells benefit from repressing PDR pump genes under normal conditions and activating them only upon exposure to xenobiotics. PDR pump substrates have been shown to induce the expression of the corresponding genes in yeast^5–8^ and animals^9^. For instance, treating rat liver cells with the MDR (multiple drug resistance) transporter substrate doxorubicin upregulates the expression of the corresponding P-glycoprotein gene^9^. 2-methyl-4-chlorophenoxyacetic has also been shown to be a substrate of yeast pleiotropic pumps (Pdr5p and Tpo1p), inducing the expression of the corresponding genes^5^. The induction of the ABC transporters genes *PDR5* and *YOR1* was observed in response to atorvastatin treatment^8^. The most striking example is cycloheximide D, an inhibitor of protein synthesis and a Pdr5p substrate^10^. At low concentrations, cycloheximide D can induce the expression of *PDR5* in yeast by binding directly to the upstream transcription factor Pdr1p^11^.

Another mechanism of pleiotropic drug resistance pumps activation that relies on mitochondria was revealed for some yeast species^12–14^. In *Saccharomyces cerevisiae*, the loss of mitochondrial DNA or the deletion of nuclear encoded genes essential for mitochondrial functioning (*e*.*g*., *FZO1*) upregulates unspecific drug resistance^15–17^. This effect requires the Pdr3p transcription factor and the components of the mitochondria-to-nucleus retrograde signalling pathway *RTG1* and *RTG2*^15^. However, as MDR pumps consume ATP, energy deprivation caused by inhibiting mitochondria can prevent the extrusion of xenobiotic agents from the cytoplasm. Indeed, the assay for MDR pump activity includes a step of energy deprivation to ensure the maximum absorption of the MDR substrates by the cells^18^.

Lipophilic cations are substrates of MDR pumps^18–20^ and a potentially promising class of molecules for pharmacological applications. Alkylated modifications of such molecules are used to produce chimeric compounds to deliver drugs to the mitochondrial matrix (*e*.*g*., antioxidants)^21,22^. Dodecyltriphenylphosphonium (C_12_TPP) has been found to act as an uncoupler, being co-transported with a free fatty acid^23^. The uncoupling activity allows to use this chemical as an anti-obesity drug in murine models^24^. Moreover, lipophilic cations including C_12_TPP appear to be competitive inhibitors of MDR^25–27^. However, whether such compounds can induce the expression of MDR genes is unclear. On the one hand, as substrates of major ABC transporters, these compounds might bind Pdr1p/Pdr3p transcription factors and activate the transcription of the transporters. Moreover, the accumulation of C_12_TPP in the mitochondria can induce the inhibition of mitochondrial enzymes^28^ or induce ROS accumulation^29^, which in turn can potentially activate the RTG-dependent activation of PDR genes. On the other hand, the sequestration of the lipophilic cations in the mitochondria may prevent their possible interactions with Pdr1p/Pdr3p transcription factors. The latter can inhibit the activation of MDR. In this work, we studied the effects of the alkylated lipophilic cation C_12_TPP on the activation of pleiotropic drug resistance system in yeast.

We found that C_12_TPP induces MDR in yeast. This activation requires the Pdr1p transcription factor, whereas the deletion of the *PDR3* gene or the genes encoding components of the mitochondria-to-nucleus retrograde signalling pathways did not abolish the induction.

## Results

**Figure 1:**
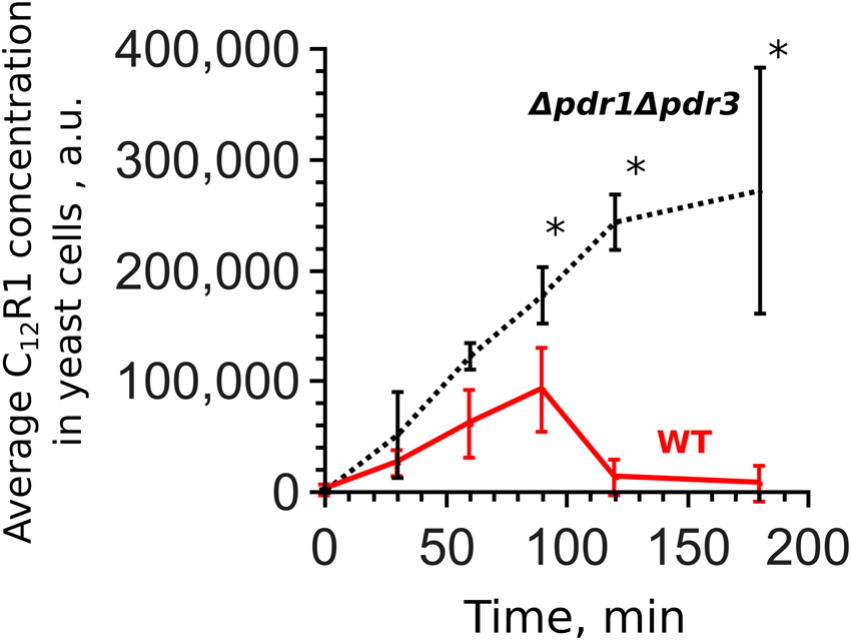
The dynamics of C_12_R1 accumulation. Yeast cells were resuspended at 2*10^4^ cells/ml in fresh YPD containing dodecylrhodamine (C_12_R1) at 200 nM. The C_12_R1 concentration was assessed using FACS (see Materials and Methods). The graphs show averages of four (WT, wild-type) or five (*Δ*pdr1*Δ*pdr3) biological replicates with standard deviations. *P = 0.017 for comparisons with WT according to the unpaired Mann-Whitney test.

We have shown recently that alkylated rhodamines are substrates of *S*. *cerevisiae* ABC transporters^27^. In a subsequent set of experiments, we observed that upon transfer from a solid medium to a liquid medium containing C_12_R1 (dodecylrhodamine 19), the cells first accumulated the dye before the amount of accumulated dye began to decrease (Fig. 1). This effect cannot be attributed to a decrease in the C_12_R1/cell ratio because there was only a modest increase in cell density during the first 180 minutes after the inoculation of yeast cells in the liquid medium (Figure S1). A lag interval preceding a decrease suggests that the observed decrease in the C_12_R1 signal could be due to the transcriptional or translational activation of pleiotropic efflux pumps. To test this possibility, we measured the dynamics of C_12_R1 accumulation in a strain with deleted *PDR1* and *PDR3* transcription factors. We did not detect any decrease in the C_12_R1 levels in this strain (Fig. 1).

**Figure 2:**
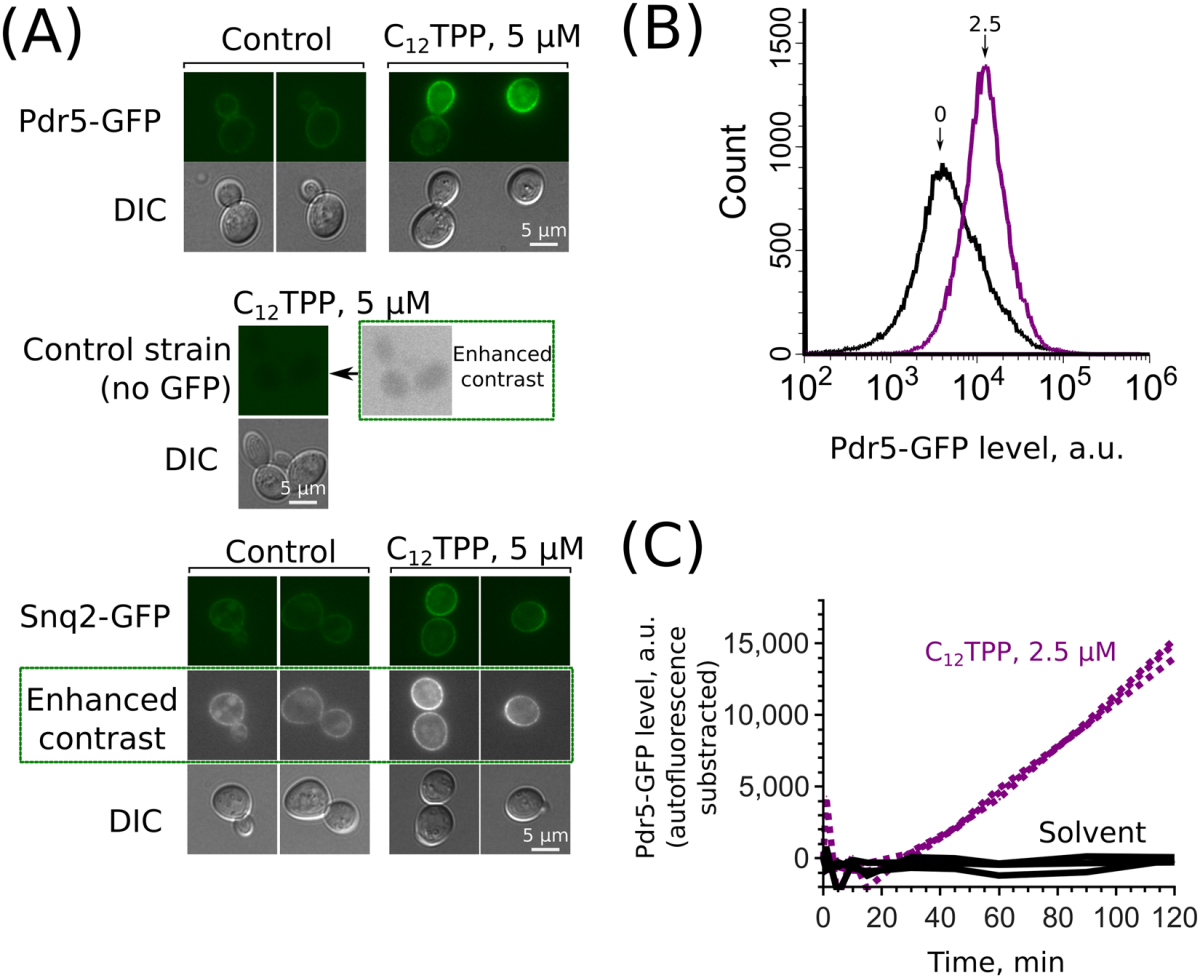
C_12_TPP induces the accumulation of the major MDR proteins Pdr5 and Snq2. (**A**) Accumulation of Pdr5-GFP and Snq2-GFP induced by C_12_TPP. The incubation time was 1 hour. All fluorescence microphotographs were taken with the same exposure time and contrast adjustments, with the exception of the ‘enhanced contrast’ panels. Green is a pseudocolour. (**B**) Representative histograms of flow cytometry experiments with yeast cells expressing Pdr5-GFP. The numbers above the arrows indicate the concentrations (µM) of supplemented C_12_TPP. The incubation time was 1 hour. (**C**) Quantification by flow cytometry of Pdr5-GFP accumulation induced by C_12_TPP. The average integral intensities of Pdr5-GFP fluorescence with the deduction of autofluorescence of GFP-negative cells were measured.

**Figure 3:**
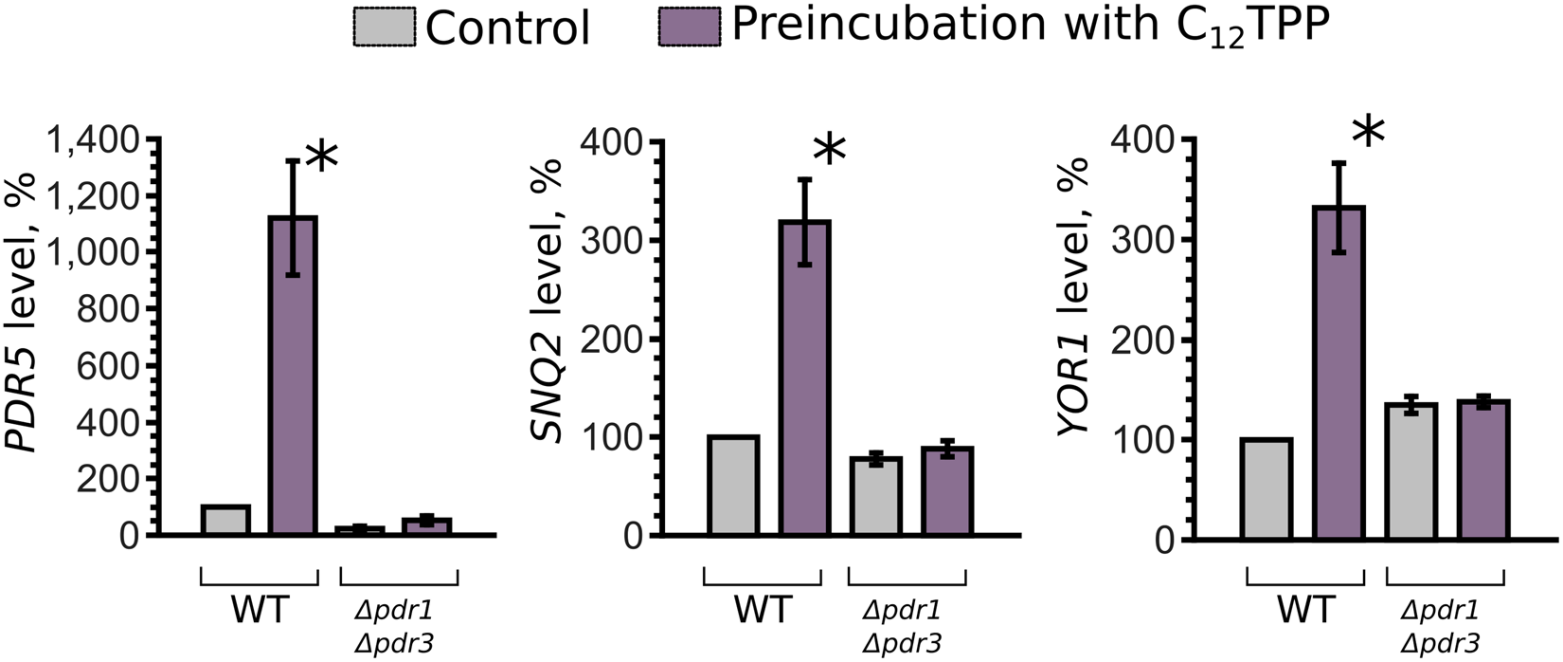
C_12_TPP (2.5 µM, 1 hours of incubation) increases the mRNA levels of *PDR genes PDR5*, *SNQ2 and YOR1*. All mRNA levels were normalized to *ACT1* levels, and the value in untreated wild-type cells (WT) was set at 100%. The bars show the mean ± standard errors, n = 4. *P = 0.028 for comparisons to an untreated control, according to the unpaired Mann-Whitney test.

As C_12_R1 fluorescence has partly overlapping emission spectra with GFP, we used the non-fluorescent lipophilic cation dodecyltriphenylphosphonium (C_12_TPP) for further experiments. We found that C_12_TPP induces an accumulation of two major pleiotropic drug resistance ABC transporters: Pdr5p and Snq2p (Fig. 2A). The signal of Yor1-GFP was relatively low (Figure S2); therefore, we could not draw any conclusions about this transporter at this stage. The increase in Pdr5-GFP levels was quantified by means of flow cytometry (Fig. 2B). In line with the C_12_R1 accumulation experiments, the Pdr5-GFP levels started to increase after approximately 30 minutes of incubation with C_12_TPP (Fig. 2C). Importantly, the C_12_TPP-induced increase in the Pdr5-GFP levels was not due to an increase in the cell volume or cell surface area, as the cell size was identical in the C_12_TPP-treated and the control yeast cells (Figure S3). Next, we measured the relative mRNA levels of three transporter genes — *PDR5*, *SNQ2* and *YOR1*— using quantitative reverse transcription PCR (RT-qPCR). We found that C_12_TPP increased the mRNA levels of these genes in the wild-type strain, while the effect in the *Δpdr1Δpdr3* strain was much less pronounced (Fig. 3). This result suggests that the C_12_TPP-induced accumulation of ABC transporters is a result of the transcriptional activation of the corresponding genes.

**Figure 4:**
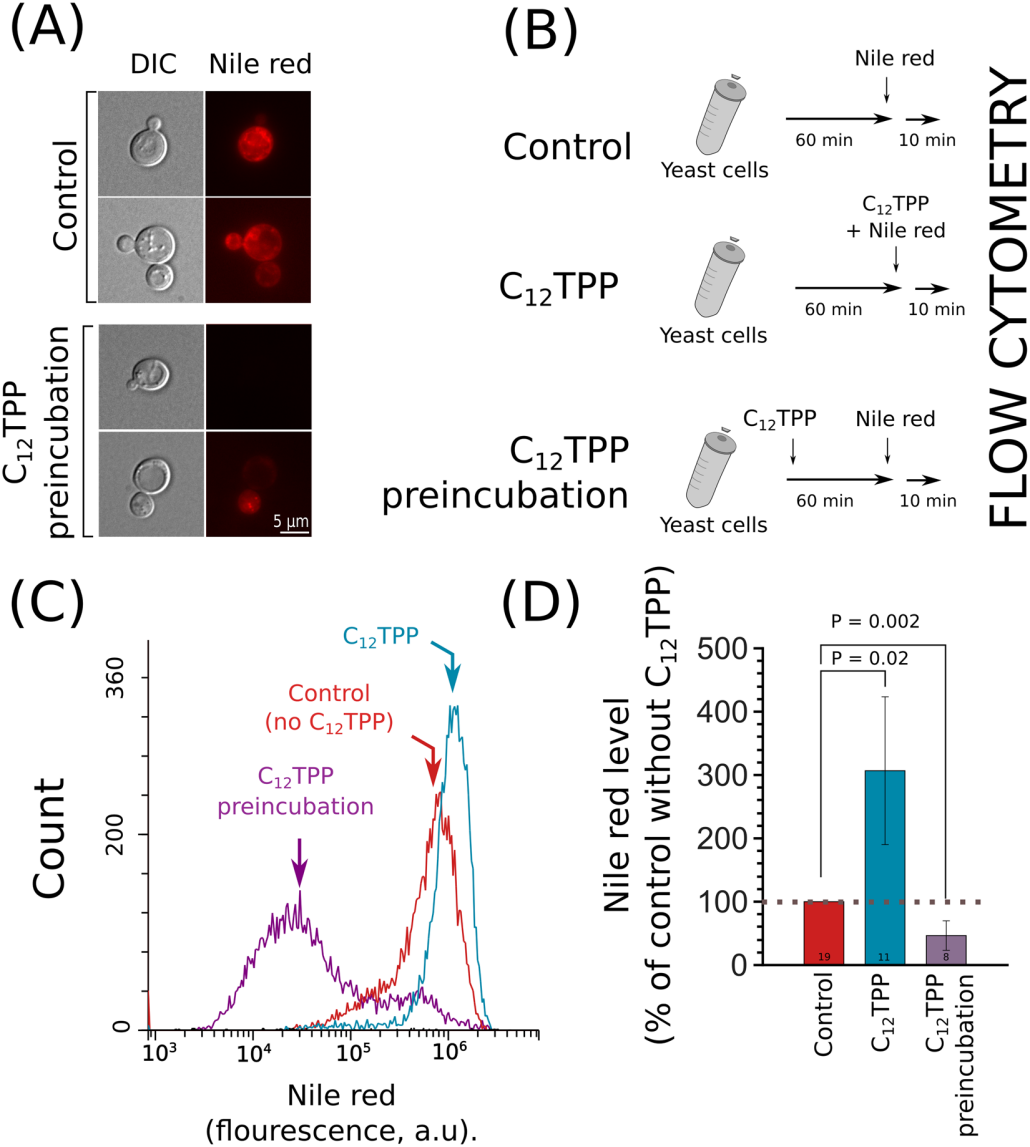
Preincubation with C_12_TPP prevents the accumulation of the MDR substrate Nile red. (**A**) Preincubation with 2.5 µM C_12_TPP for 1 hour decreases the accumulation of Nile red in yeast cells. Red is a pseudocolour. (**B**) The design of the experiment. (**C**) Representative histograms of flow cytometry experiments of Nile red accumulation in yeast cells. C_12_TPP (2.5 µM) was added either before or simultaneously with Nile red (3.5 µM). (**D**) Quantification of flow cytometry results (average ± standard deviation). Here, 100% corresponds to the average fluorescence (level) of Nile red in the control samples. The numbers of separate experiments are indicated below the bars. P values were calculated for comparison with the untreated control (WT) according to the unpaired Mann-Whitney test with Bonferroni adjustments.

Does the accumulation of ABC proteins induced by C_12_TPP indeed lead to the increased efflux of the transporter substrates? To answer this question, we studied the effect of preconditioning with C_12_TPP on the accumulation of the ABC transporter non-charged fluorescent substrate Nile red. This compound is a substrate of pleiotropic transporters of *Candida albicans*^30^. We found that *SNQ2* gene overexpression significantly decreased the accumulation of Nile red in *S*. *cerevisiae cells*, while its repression had the opposite effect (Figure S4). The repression of the ABC transporter gene *PDR5* also increased the accumulation of Nile red in the cells (Figure S4). We found that the preincubation of yeast cells with C_12_TPP inhibited Nile red accumulation in cells (Fig. 4A–D). At the same time, in line with our previous study^26^, we noticed that the simultaneous supplementation of Nile red and C_12_TPP to yeast incubation media facilitated the uptake of Nile red by cells (Fig. 4B–D). Thus, C_12_TPP plays both inhibitory and activating roles in MDR regulation, and the contribution of each role changes with the duration of cellular exposure to the chemical.

**Figure 5:**
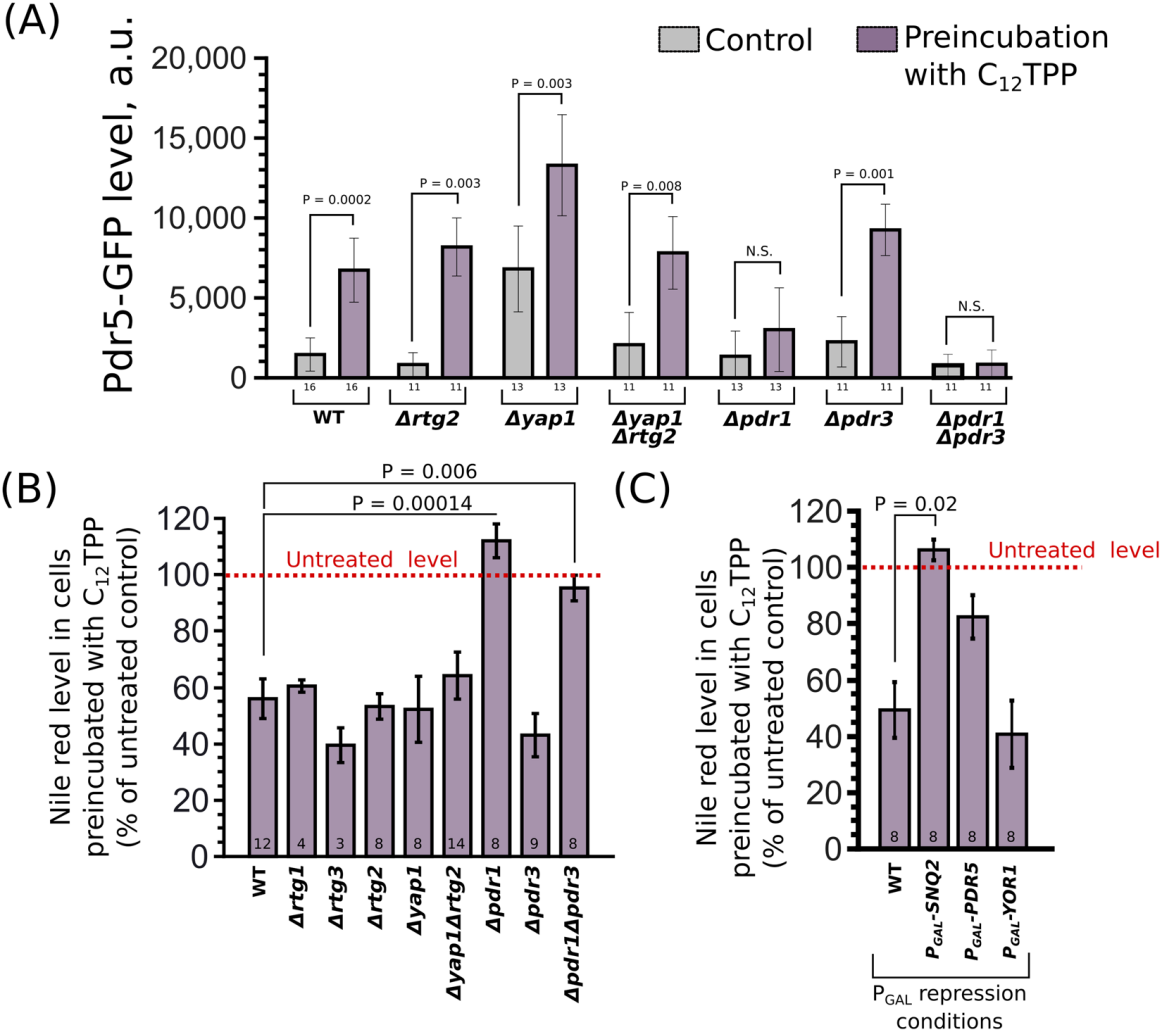
The role of retrograde signalling and PDR genes in the C_12_TPP-induced activation of PDR. (**A**) Pdr5-GFP levels in knockout yeast strains grown in YPD and treated with C_12_TPP (2.5 µM, 1 hour). (**B**) C_12_TPP-induced (2.5 µM, 1 hour) decreases in Nile red levels in the knockout yeast strains. (**C**) C_12_TPP-induced (2.5 µM, 1 hour) decreases in Nile red levels in yeast strains with repressed ABC transporter genes *SNQ2*, *PDR5* or *YOR1*. (**A**–**C**) Cells were grown in YPD medium (the condition of P_GAL_ repression). Data are shown as the mean ± standard error. Numbers of biological replicates are indicated below the bars. P values were calculated for comparisons with the untreated control (WT) according to unpaired Mann-Whitney test with Bonferroni adjustments for multiple comparisons.

What is the mechanism of C_12_TPP-dependent MDR upregulation? The lipophilic cations can inhibit respiration^28^ and facilitate proton leakage^23,31^. At the same time, dysfunctional mitochondria upregulate mitochondria-to-nucleus signalling mediated by Rtg-proteins, and such signalling has been shown to upregulate *PDR5*^15^. Thus, we tested the role of the retrograde pathway in C_12_TPP-induced resistance using the non-charged lipophilic fluorescent dye Nile red. The inhibition of the pathway by *RTG2* knockdown did not prevent the C_12_TPP-induced increase in Pdr5-GFP levels in yeast cells (Fig. 5A). The release of mitochondrially produced hydrogen peroxide into the cytoplasm is another possible signalling pathway from dysfunctional mitochondria to the nucleus (for review, see^32^). This pathway relies on cytoplasmic Yap1p^33^, the transcription factor activated under oxidative stress^34^. While the primary targets of Yap1p are antioxidant genes^35^_,_ Yap1p may also contribute to the activation of PDR genes^36^. To test the role of Yap1p in the C_12_TPP activation of MDR, we produced the *Δyap1 Pdr5-GFP* strain. Taking into account the possible crosstalk between the Yap1p and Rtg pathways,^33^ we tested the activation in the double *Δyap1Δrtg2* mutant. We found that C_12_TPP was capable of upregulating the Pdr5-GFP levels in all tested strains with disrupted mitochondria-to-nucleus signalling (Fig. 5A). Thus, we concluded that *YAP1* does not play a role in C_12_TPP-induced PDR. Next, we tested the contribution of the transcription factors *PDR1* and *PDR3* in the C_12_TPP-dependent upregulation of Pdr5-GFP levels. We found that the deletion of *PDR1* but not *PDR3* inhibited this effect (Fig. 5A). The accumulation of Snq2-GFP was also abolished in the *Δpdr1* and *Δpdr1Δpdr3* strains (Figure S5). Likewise, C_12_TPP was able to induce a decrease in Nile red levels in the *Δyap1*, *Δrtg1*, *Δrtg3*, *Δrtg2*, *Δyap1Δrtg2*, and *Δpdr3* strains but displayed no effect if the *PDR1* gene was deleted (Fig. 5B). We also showed that repressing the *SNQ2* gene prevented the C_12_TPP-induced decrease in Nile red accumulation (Fig. 5C). This result suggests that the Snq2p ABC transporter is involved in the C_12_TPP-mediated induction of MDR. However, the repression of PDR genes affects the accumulation of both C_12_TPP (inducer) and Nile red (sensor). Therefore, the repression of a transporter gene can increase C_12_TPP concentrations in the cells and, in this way, facilitate a stronger compensatory response by other genes. Thus, the negative result with the *PDR5* and *YOR1* genes does not exclude the possibility that these genes contribute to C_12_TPP-mediated MDR activation. Notably, the overexpression of *PDR1* targets *SNQ2* and *PDR5* abolished the effect of C_12_TPP on Nile red accumulation (Figure S6). This observation suggests that the lipophilic cation needs to accumulate inside the cells to activate MDR. Alternatively, ABC transporter overexpression can be sufficient to drive the maximal efflux rate. However, we believe the latter explanation is unlikely because the addition of C_12_TPP to the wild-type cells lowered the Nile red levels much more than in the P_GAL_ strains.

**Figure 6:**
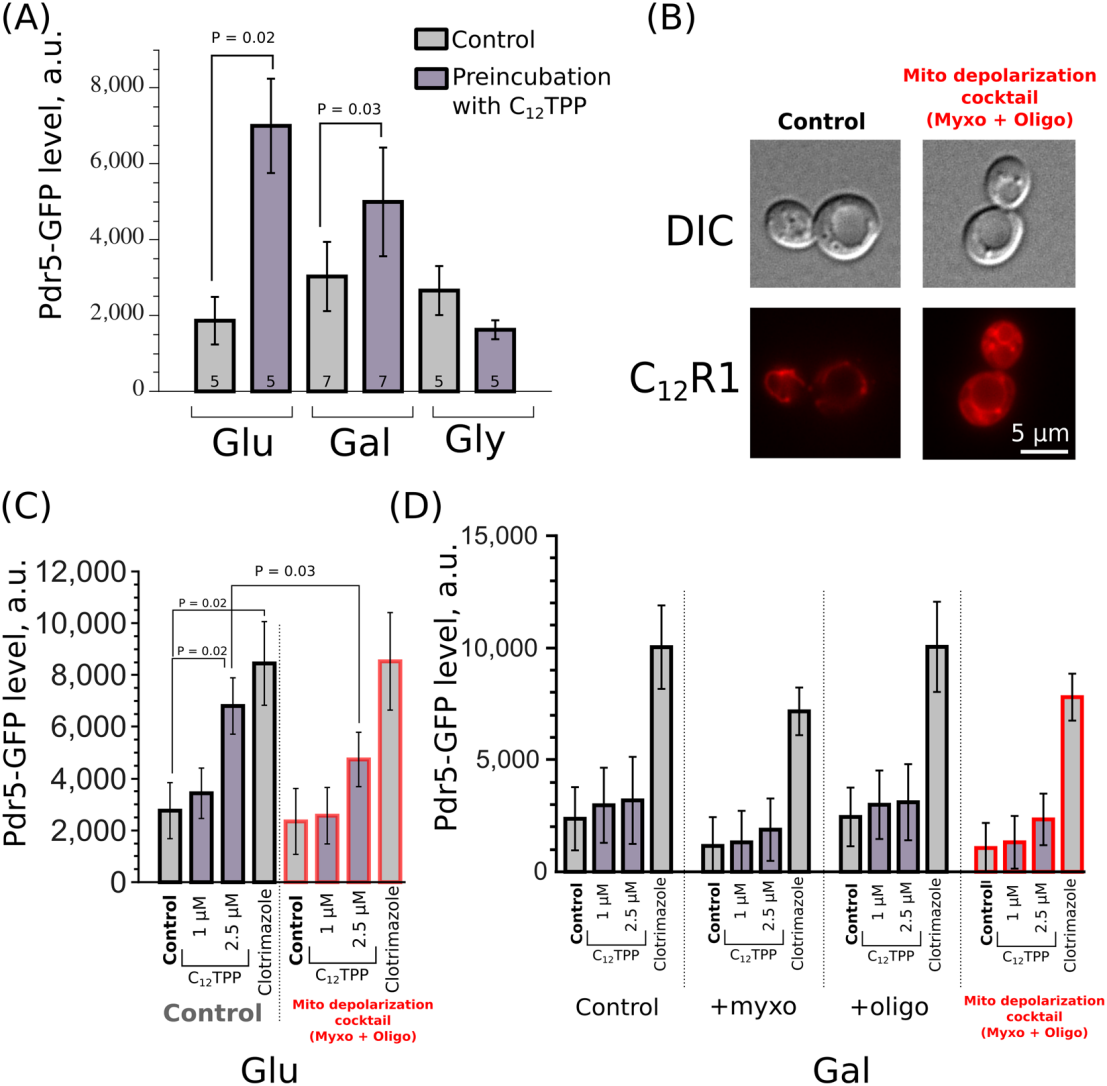
Mitochondrial depolarization does not facilitate C_12_TPP-induced Pdr5-GFP accumulation. (**A**) The effect of a carbon source on the C_12_TPP-induced (2.5 µM, 1 hour) change in the Pdr5-GFP level. (**B**) Myxothiazol at 7 µM and oligomycin A at 5 µg/ml admixture increases the cytoplasmic (diffuse) signal from the lipophilic cation C_12_R1 (200 nM) in wild-type yeast cells. Red is a pseudocolour. (**C**,**D**) Quantification of Pdr5-GFP level in cells (mean ± standard deviation) from flow cytometry experiments with myxothiazol and/or oligomycin A for yeast cells grown in YPD (C, n = 5) or YPGal (D, n = 6). Clotrimazol was added to a final concentration of 20 µM, with a 1-hour incubation time. P values were calculated according to a Wilcoxon signed-rank test with Bonferroni adjustments.

Lipophilic cations, as positively charged molecules, accumulate in negatively charged cellular organelles — mitochondria^37^. We expected that the accumulation of such compounds in the mitochondria would mitigate the interaction of the cations with their sensors in the cytoplasm. To test this, we compared the activation of MDR in different growth media. In the presence of high glucose concentrations, yeast cells suppress mitochondrial functions, while in the presence of a poorly fermentable carbon source (galactose) or non-fermentable carbon source (glycerol), yeast cells rely on mitochondrial energetics^38^. We tested the effect of C_12_TPP on Pdr5-GFP levels in yeast cells in rich medium supplemented with either galactose or glycerol. We found that the inoculation of yeast cells in YPGal medium supplemented with C_12_TPP triggered the accumulation of Pdr5-GFP. At the same time, there was no increase in Pdr5-GFP levels in the glycerol (Fig. 6A). The uncoupling^23,31^ or inhibitory^28^ effects of C_12_TPP in the mitochondria may deplete cellular ATP and prevent Pdr5-GFP protein synthesis. In agreement with this hypothesis, myxothiazol (an inhibitor of the mitochondrial coenzyme Q - cytochrome *c* reductase) abolished the induction of Pdr5p-GFP upregulation induced by azole antifungal clotrimazole in glycerol-based medium (Figure S7).

Can mitochondrial depolarization increase the C_12_TPP-dependent activation of PDR in our experimental model? This would be possible if the equilibration in the lipophilic cation concentrations between the cell and incubation medium was relatively slow (Fig. 1). Thus, the intracellular distribution of the cation is likely to affect the detection of the molecule. If so, an inhibition of the respiratory chain can, due to mitochondrial depolarization, prevent accumulation of the lipophilic cation in the mitochondrial matrix, subsequently increasing the activation of MDR. First, we tested whether the inhibition of the respiratory chain by myxothiazol increases the cytoplasmic levels of the fluorescent lipophilic cation C_12_R1. For glucose-supplemented rich medium, we used a mixture of myxothiazol and an inhibitor of ATP-synthase, oligomycin A, to prevent the energization of mitochondria by ATP hydrolysis driven by mitochondrial ATP-synthase. We used a concentration of the inhibitors sufficient to inhibit the growth rate of the control laboratory strain in the presence of a non-fermentable carbon source^39^. We found that the mixture of the inhibitors (“depolarization cocktail”) increased the cytoplasmic signal of C_12_R1 in yeast cells (Fig. 6B). However, the mitochondrial depolarization did not upregulate the C_12_TPP-induced increase in Pdr5-GFP levels in either glucose-based (Fig. 6C) or galactose-based medium (Fig. 6D). We suggested that the addition of 2.5 µM of C_12_TPP causes the maximum possible activation of Pdr5-GFP expression; therefore, we tested lower concentrations of the chemical. However, there was no significant increase in Pdr5-GFP levels in the presence of 1 µM C_12_TPP. As oligomycin inhibits Pdr5p^40^ and, therefore, may interfere with the compensatory activation of PDR genes, we tested the C_12_TPP-induced Pdr5-GFP accumulation in the cells treated with sodium azide. Sodium azide inhibits respiration as well as ATPase activity. In line with our previous results, C_12_TPP did not activate MDR in the presence of NaN_3_ (Figure S8). Together, these data suggest that mitochondrial depolarization does not facilitate lipophilic cation-dependent MDR activation or that this activation takes place at a relatively narrow range of the conditions.

## Discussion

The exposure of yeast cells to xenobiotics favours the selection of cells with upregulated PDR gene expression^41^. Accordingly, the activation of pleiotropic drug resistance transporter genes decreases the efficiency of azole antifungal treatments^42^. Therefore, the inhibition of the PDR transporters appears to be a promising strategy to increase the treatment efficiency of emerging multiple drug resistant pathogenic fungal strains. Some cationic amphiphilic compounds, the substrates of PDR transporters, were shown to sensitize yeast cells to azole antifungals^26,27,43–45^. However, the accumulation of an MDR substrate in the cell upregulates the expression of PDR transporters genes^5,11^, which can significantly limit the application of this approach. At the same time, the mechanism of PDR upregulation is not absolutely clear.

In our study we tested the ability of the lipophilic cation C_12_TPP to activate multiple drug resistance in *Saccharomyces cerevisiae*. We expected that as a positively charged molecule, C_12_TPP could accumulate in the mitochondria and thus evade sensing by the cytoplasmic/nuclear transcription factors *PDR1* and *PDR3*. However, C_12_TPP induced an increase in the levels of both *PDR5* mRNA and Pdr5-protein and, at the same time, prevented the accumulation of Nile red, the neutral fluorescent substrate of PDR transporters (Figs 4 and 5). Moreover, the depolarization of the mitochondria, which inhibits sequestration of C_12_TPP inside the mitochondria, did not upregulate C_12_TPP-induced MDR activation (Fig. 6). At the same time, the presence of the functional transcription factor gene *PDR1* was necessary for the effects of C_12_TPP (Fig. 5). We speculate that there is a concentration window for each toxic chemical within which the chemical upregulates cellular protection mechanisms. This window is limited from above by the energetic capacity of the cell, i.e., the excess amount of a metabolic inhibitor can prevent the protective response at the transcriptional or translational level. From below, the window is limited by the specificity of the sensing factors. Thus, the accumulation in the mitochondria may prevent the activation of MDR by C_12_TPP in a relatively narrow concentration range. Although this possibility cannot be excluded with our experiments, we have shown that mitochondrial depolarization does not induce the accumulation of Pdr5-GFP in yeast cells in the presence of relatively low (1 µM) concentrations of C_12_TPP (Fig. 6C,D). Importantly, we proved that under the same conditions (the combination of respiratory chain inhibitor myxothiazol and inhibitor of ATP-synthase oligomycin A), the azole antifungal clotrimazole still induced Pdr5-GFP accumulation (Fig. 6C,D).

How can a cell sense the xenobiotics that accumulate in the mitochondria? One of the known mechanisms of PDR activation relies on direct interaction with the transcription factor Pdr1p^11^. Therefore, the binding to the transcription factor competes with the mitochondrial uptake of the lipophilic cation. If the affinity of Pdr1p to C_12_TPP is sufficiently high, the Pdr1p targets will be activated even in the presence of highly energized mitochondria. However, we think that this explanation is unlikely because C_12_TPP in low concentrations is not able to induce MDR (Fig. 6C,D). Alternatively, the substrates of MDR can indirectly activate *PDR1* by inhibiting the efflux of endogenous substrates of PDR transporters. Such substrates might activate the expression of the transporters by the aforementioned mechanism. There are data suggesting that Pdr5p and Snq2p can export a metabolite that acts as a quorum-sensing factor in yeast suspension cultures^46,47^. In this case, the cellular localization of C_12_TPP could play a minor role because the insufficient activity of MDR will cause accumulation of the endogenous metabolites, thus activating MDR.

Another possible way of sensing the xenobiotics is to measure some vital cellular metabolites or the energy level. For instance, Rtg2p binding to downstream components of the retrograde signalling cascade Mks1p is regulated by the ATP level^48^. Thus, the retrograde signalling activation of PDR genes may integrate different pathways via the ATP level. However, in our experiments, the deletion of *RTG2* did not prevent the C_12_TPP-induced activation of Pdr5-GFP accumulation.

To summarize, we have shown that the penetrating lipophilic cation C_12_TPP upregulates pleiotropic drug resistant ABC transporters in yeast *Saccharomyces cerevisiae*. The activation mechanism includes the transcription factor *PDR1* but not *PDR3, RTG2* or *YAP1*. Mitochondrial depolarization induced by the uncouplers did not facilitate C_12_TPP-induced multiple drug resistance activation. Taken together, our data suggest that the sensing of pleiotropic drug resistance in yeast cells is robust to variations in xenobiotic localization.

## Materials and Methods

### Strains and growth conditions

In this study, we used strains with *W303* or *BY4741* genetic backgrounds. Their derivatives are listed in Table 1. In the mutant strains, the genomic copies of ABC transporter genes were under the control of P_GAL_. The P_GAL_ promoter was induced by the supplementation of galactose and repressed by glucose. To delete the complete open reading frames, we amplified the deletion cassettes from Invitrogen yeast deletion collection strains with the gene-specific primers. Double and triple mutants were produced by crossing the corresponding single mutants and subsequent tetrad dissection. All strains were verified by PCR with primers that were able to yield product only with the correct insertions. *PDR5-GFP*, *SNQ2-GFP* and *YOR1-GFP* strains were from Invitrogen GFP collection^49^. Rich growth medium YPD (yeast peptone D-glucose), YPRafGal (yeast peptone raffinose galactose), YPGal (yeast peptone galactose) and YPGly (yeast peptone glycerol) were prepared according to Sherman^50^. Yeast extract was obtained from BD; bactoagar, bactopeptone and D-glucose were from Amresco. Dodecyltriphenylphosphonium (C_12_R1) and dodecylrhodamine 19 (C_12_R1) were previously synthesized in our institute as bromide salts^51,52^, and the other chemicals were obtained from Sigma-Aldrich.

**Table 1:** Strains used in the study.

Strain	Genotype	Parental strains and/or references
*W303-1A*	*MATa ade2-101 his3-11 trp1-1 ura3-52 can1-100 leu2-3*	Laboratory of A. Hyman
*Δpdr1* ^*a*^	*MATa ade2-101 his3-11 trp1-1 ura3-52 can1-100 leu2-3 pdr1Δ::KanMX4*	*W303-1A*
*Δpdr3* ^*a*^	*MATalpha ade2-101 his3-11 trp1-1 ura3-52 can1-100 leu2-3 pdr3Δ::KanMX4*	*W303-1B*
*Δpdr1Δpdr3* ^*b*^	*MATa ade2-101 his3-11 trp1-1 ura3-52 can1-100 leu2-3 pdr1Δ::KanMX4 pdr3Δ::KanMX4*	*Δpdr1*, *Δpdr3*
*Δrtg1*	*MATa ade2-101 his3-11 trp1-1 ura3-52 can1-100 leu2-3 rtg1Δ::KanMX4*	*Zyrina et al*.^33^
*Δrtg2*	*MATa ade2-101 his3-11 trp1-1 ura3-52 can1-100 leu2-3 rtg2Δ::KanMX4*	*Zyrina et al*.^33^
*Δrtg3*	*MATa ade2-101 his3-11 trp1-1 ura3-52 can1-100 leu2-3 rtg3Δ::KanMX4*	*Zyrina et al*.^33^
*Δyap1*	*MATalpha ade2-101 his3-11 trp1-1 ura3-52 can1-100 leu2-3 yap1Δ::KanMX4*	*Zyrina et al*.^33^
*Δrtg2Δyap1*	*MATa ade2-101 his3-11 trp1-1 ura3-52 can1-100 leu2-3 rtg2Δ::KanMX4 yap1Δ::KanMX4*	*Zyrina et al*.^33^
*Pdr5-GFP*	*MATa his3Δ1 leu2Δ0 met15Δ0 ura3Δ0 PDR5-GFP::HIS3*	*Huh et al*.^49^
*Snq2-GFP*	*MATa his3Δ1 leu2Δ0 met15Δ0 ura3Δ0 SNQ2-GFP::HIS3*	*Huh et al*.^49^
*Snq2-GFP Δpdr1Δpdr3* ^*b*^	*MATa ade2-101 his3 leu2 met15Δ0 ura3 trp1-1 can1-100 SNQ2-GFP::HIS3 pdr1Δ::KanMX4 pdr3Δ::KanMX4*	*SNQ2-GFP*, *Δpdr1Δpdr3*
*Snq2-GFP Δpdr1* ^*b*^	*MATa ade2-101 his3 leu2 met15Δ0 ura3 trp1-1 can1-100 SNQ2-GFP::HIS3 pdr1Δ::KanMX4*	*SNQ2-GFP*, *Δpdr1Δpdr3*
*Snq2-GFP Δpdr3* ^*b*^	*MATa ade2-101 his3 leu2 met15Δ0 ura3 trp1-1 can1-100 SNQ2-GFP::HIS3 pdr3Δ::KanMX4*	*SNQ2-GFP*, *Δpdr1Δpdr3*
*P*_*GAL*_ *-SNQ2*	*MATa ade2-101 his3-11 trp1-1 ura3-52 can1-100 leu2-3 PGAL-SNQ2::HIS3*	*Knorre et al*.^26^
*P*_*GAL*_ *-PDR5*	*MATa ade2-101 his3-11 trp1-1 ura3-52 can1-100 leu2-3 PGAL-PDR5::HIS3*	*Knorre et al*.^26^
*Pdr5-GFP Δpdr1Δpdr3* ^*b*^	*MATa ade2-101 his3 leu2 met15Δ0 ura3 trp1-1 can1-100 PDR5-GFP::HIS3 pdr1Δ::KanMX4 pdr3Δ::KanMX4*	*Pdr5-GFP*, *Δpdr1Δpdr3*
*Pdr5-GFP Δpdr1* ^*a*^	*MATa his3Δ1 leu2Δ0 met15Δ0 ura3Δ0 PDR5-GFP::HIS3 pdr1Δ::KanMX4*	*Pdr5-GFP*
*Pdr5-GFP Δpdr3* ^*a*^	*MATa his3Δ1 leu2Δ0 met15Δ0 ura3Δ0 PDR5-GFP::HIS3 pdr3Δ::KanMX4*	*Pdr5-GFP*
*Pdr5-GFP Δrtg2* ^*b*^	*MATa ade2-101 his3 leu2 met15Δ0 ura3 trp1-1 can1-100 PDR5-GFP::HIS3 rtg2Δ::KanMX4*	*Pdr5-GFP*, *Δrtg2*
*Pdr5-GFP Δyap1* ^*b*^	*MATa ade2-101 his3 leu2 met15Δ0 ura3 trp1-1 can1-100 PDR5-GFP::HIS3 yap1Δ::KanMX4*	*Pdr5-GFP*, *Δyap1*
*Pdr5-GFP Δrtg2Δyap1* ^*b*^	*MATa ade2-101 his3 leu2 met15Δ0 ura3 trp1-1 can1-100 PDR5-GFP::HIS3 rtg2Δ::KanMX4 yap1Δ::KanMX4*	*Pdr5-GFP Δrtg2*, *Δyap1*
*Yor1-GFP*	*MATa his3Δ1 leu2Δ0 met15Δ0 ura3Δ0 YOR1-GFP::HIS3*	*Huh et al*.^26^

^a^Strain produced by the transformation of the PCR cassette.

^b^Strain produced by crossing and tetrad dissection.

### Fluorescent microscopy

To study the accumulation of GFP proteins, we used the fluorescence microscope Olympus BX41 with the U-MNIBA3 (excitation wavelength 470–495 nm; beamsplitter filter 505 nm; emission 510–550 nm) filter set. The accumulation of C_12_R1 and Nile red was visualized with the U-MNG2 (excitation 530–550 nm, beamsplitter filter 570 nm; emission >590 nm) filter set. All results were reproduced in at least three biological replicates.

### Flow cytometry

Fluorescence of GFP was assessed with a CytoFlex (Beckman) flow cytometer using an excitation wavelength of 488 nm on the emission filter (525/40 nm). The accumulation of Nile red was measured with an emission filter (585/42 nm). Bar plots represent the average of averages of cell populations from separate biological experiments. At least 10 000 events were analysed in each experiment.

### Quantitative reverse transcription PCR (RT-qPCR) analysis

RNA was isolated from yeast cells using the hot formamide extraction method described in^53^. RNA samples were isolated independently four times on separate days. RNA quality and quantity were assessed by electrophoresis and spectrophotometry. cDNA was synthesized by annealing 2 μg of RNA with 0.1 μg of random hexamers and 0.1 μg of Oligo-dT using Superscript III reverse-transcriptase (Thermo Fisher Scientific) for 1 hour at 44 °C. RT-qPCR was carried out using the CFX96 Touch™ Real-Time PCR Detection System (Bio-Rad, Hercules, CA, USA). We used primer sequences for PDR genes^54^. For the detection of the target genes, the Eva Green master mix (Syntol, Russia) was used according to the manufacturer’s instructions. The thermal profile for EVA Green RT-qPCR included an initial heat-denaturing step at 95 °C for 3 minutes, 40 cycles at 95 °C for 15 sec, an annealing step for 30 sec and 72 °C for 30 sec, coupled with fluorescence measurements. Following amplification, the melting curves of PCR products were monitored to determine the specificity of the amplification. Each sample was analysed in triplicate, and a non-template control was added to each run. The PCR efficiency (E) was calculated according to the equation E = 10(−1/slope) by performing the standard curves. Target mRNA levels were normalized to the reference gene *ACT1*.

### GFP accumulation assay

Cells with GFP expression were grown overnight in solid medium (YPD, YPGal or YPGly) and then resuspended to a density of 2*10^4^ cells/ml in the same medium. Fluorescence was assessed with a flow cytometer after 1 hour of preincubation with the inhibitors (or solvent) at 30 °C.

### Nile red accumulation assay

To assess Nile red accumulation, we grew yeast cells overnight in the corresponding rich growth medium and resuspended the cells to a density of 2*10^4^ cells/ml in the same medium. After 1 hour of preincubation with the inhibitors (or solvent) at 30 °C, Nile red was added to a final concentration of 3.5 μM. After a 10-minute incubation at 30 °C, the amount of accumulated Nile red in the yeast cells was measured by flow cytometry.

### Data analysis

Data were analysed with a non-parametric Wilcoxon-Mann-Whitney test using R. The Bonferroni adjustment for multiple comparisons was applied where appropriate.

### Data availability

The datasets generated and/or analysed during the current study are available from the corresponding author on reasonable request.

## Electronic supplementary material

Supplementary data
