# Supplementary material for: Penetrating cations induce pleiotropic drug resistance in yeast
Source: Sci Rep. 2018 May 25;8:8131. doi: 10.1038/s41598-018-26435-z (PMC5970188; doi:10.1038/s41598-018-26435-z)
Supplement: Supplementary file 1 — Supplementary data [file 41598_2018_26435_MOESM1_ESM.pdf]

SUPPLEMENTARY MATERIALS

Penetrating cations induce pleiotropic drug resistance in yeast

Kseniia V. Galkina[1], Elizaveta G. Besedina[1], Roman A. Zinovkin[2,3,4], Fedor F. Severin[2,3], Dmitry A. Knorre[2,4*]

[1]Faculty of Bioengineering and Bioinformatics, Moscow State University, Leninskiye Gory 1-73, Moscow 119991, Russia

[2]Belozersky Institute of Physico-Chemical Biology, Moscow State University, Leninskiye Gory 1-40, Moscow 119991, Russia

[3]Institute of Mitoengineering, Moscow State University, Leninskiye Gory 1, Moscow 119991, Russia

[4]Institute of Molecular Medicine, Sechenov First Moscow State Medical University, Moscow 119991, Russia

* knorre@belozersky.msu.ru

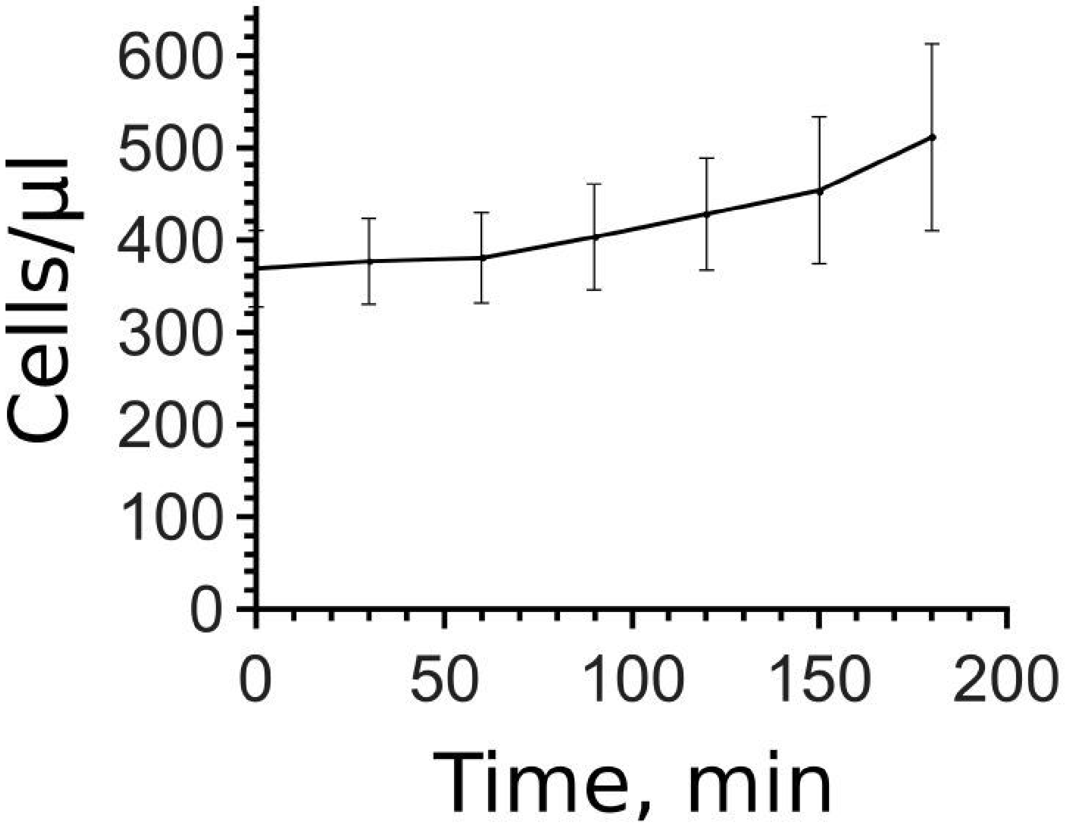

Figure S1. Concentration of the control W303 cells in rich liquid medium within first 90 minutes after inoculation measured with flow cytometer. Mean ± SD is indicated (n = 3).

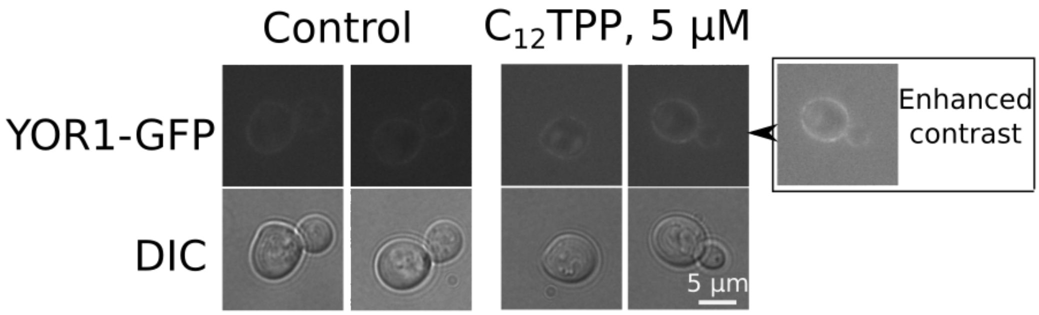

Figure S2. Yor1-GFP levels in the presence and in the absence of of 5 µM C_{12}TPP. Incubation time 1 hour. All fluorescent microphotographs were taken with the same exposure time and contrast adjustments with the exception of ‘enhanced contrast’ panel.

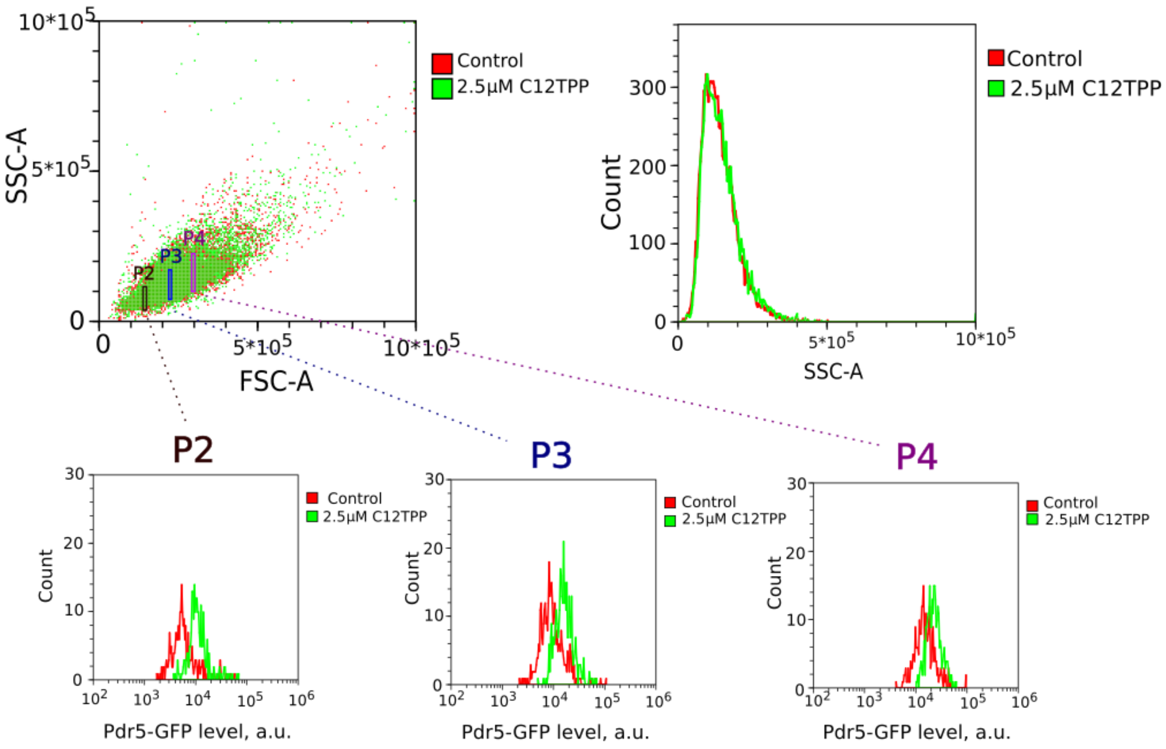

Figure S3. C_{12}TPP (2.5 μM, incubation time 1 hour) does not induce an increase in yeast cell size (forward and side scattering, upper panels). The increase in Pdr5-GFP levels can be observed in different cell-size gates (lower panels).

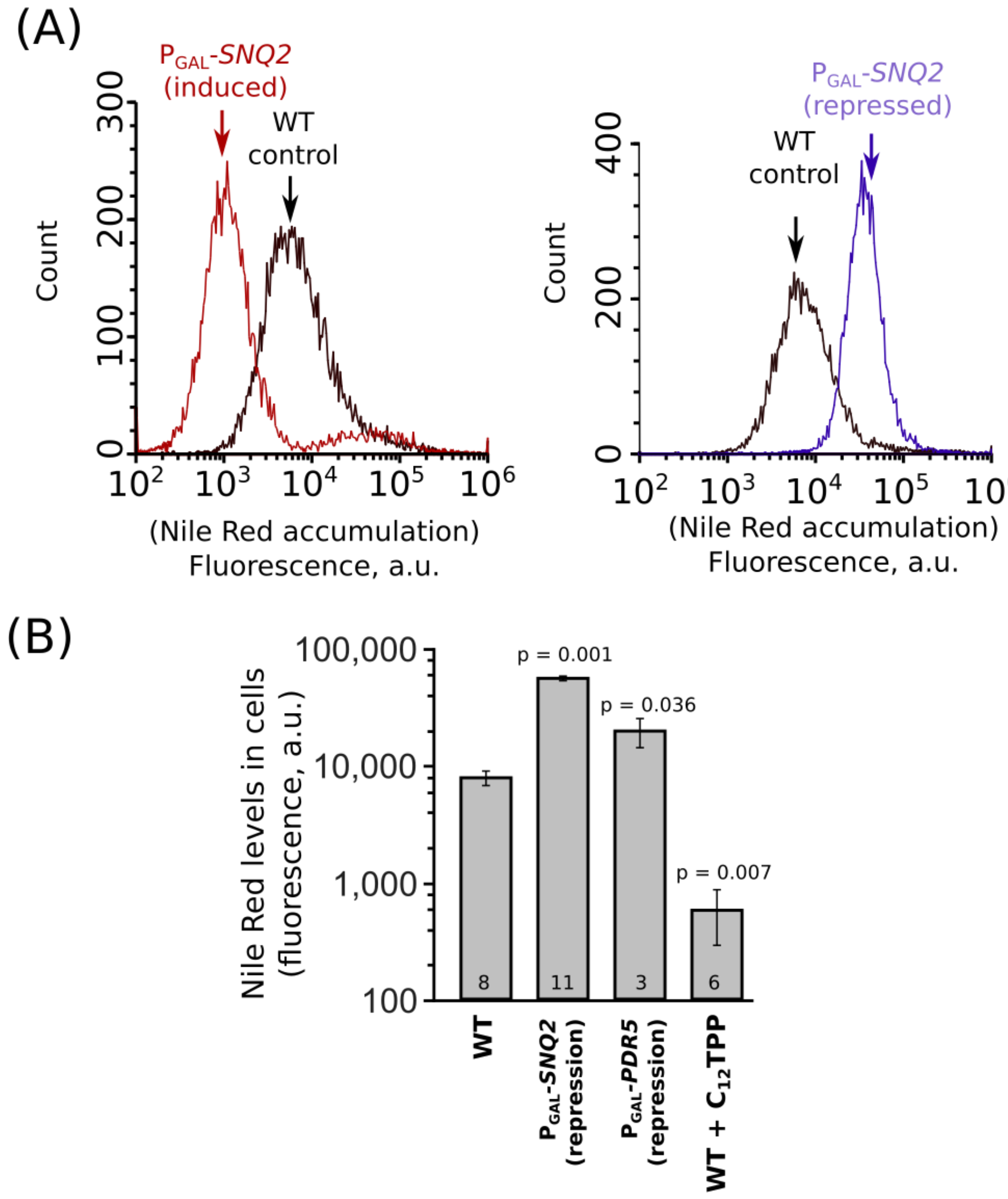

Figure S4. Nile red accumulation in yeast cells is affected by the expression level of PDR transporters. (A) Representative result of flow cytometry experiment with Nile red in wild type or P_{GAL}-SNQ2 yeast cells pregrown on YPRafGal (left) or YPD (right). Incubation time for this experiment was 1 hour. Quantification of the flow cytometry analysis for cells grown in YPD (B). Numbers of separate experiments are indicated below the bars, P values were calculated for the comparisons with untreated W303 control (WT) according to unpaired Mann-Whitney test with Bonferroni adjustments.

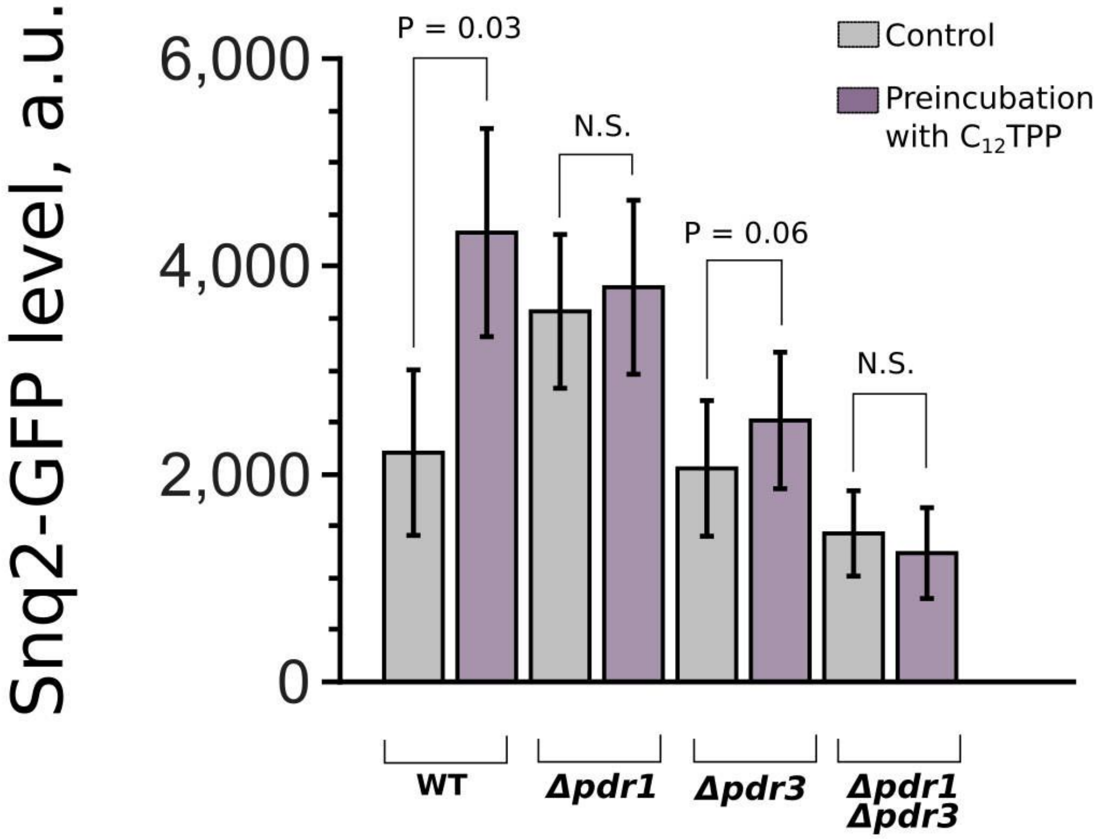

Figure S5. C_{12}TPP-induced increase of Snq2-GFP levels in the knockout yeast strains (mean ± standard deviation, n = 8). P values were calculated according to Wilcoxon signed-rank test with Bonferroni adjustments.

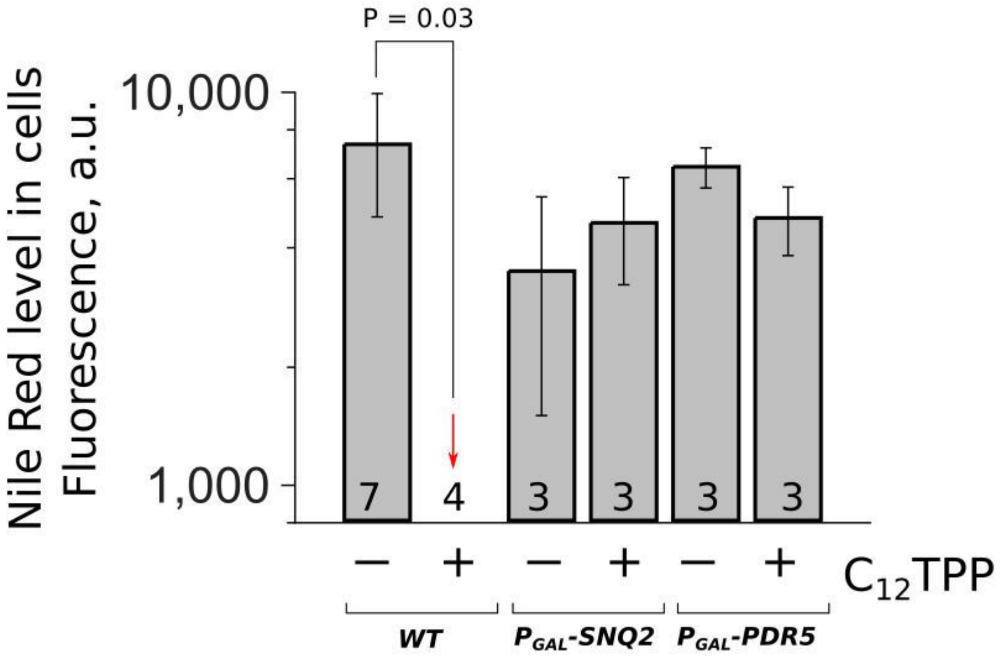

Figure S6. C_{12}TPP-induced decrease of Nile red levels in yeast strains with upregulated ABC-transporter genes *SNQ2* or *PDR5*. Yeast cells were grown in YPRafGal medium.

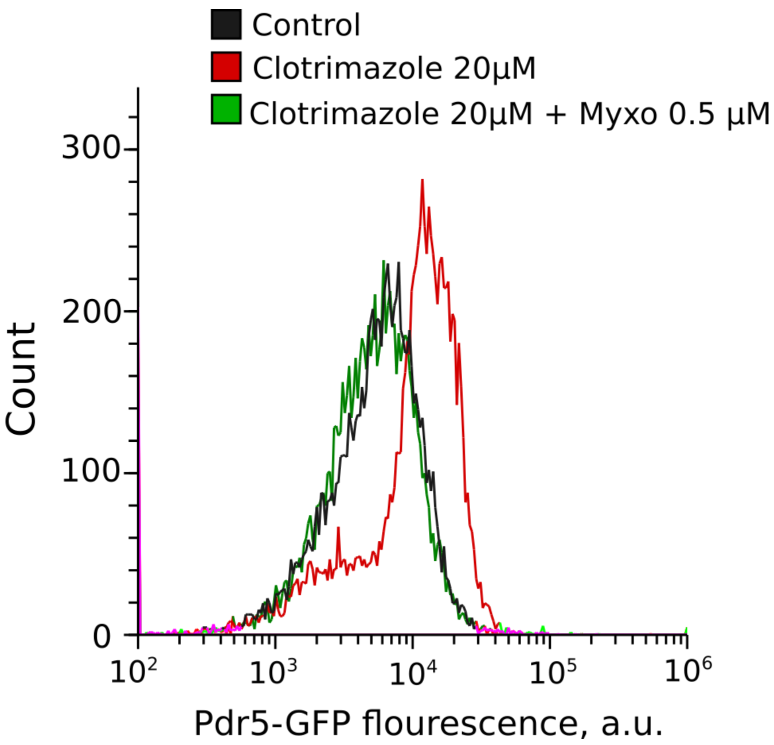

Figure S7. Clotrimazole (20 μM, incubation time 1 hour) induced increase of Pdr5-GFP levels in glycerol based medium can be inhibited by adding respiratory chain inhibitor myxothiazol (0.5 μM). The result of a typical experiment (n=3).

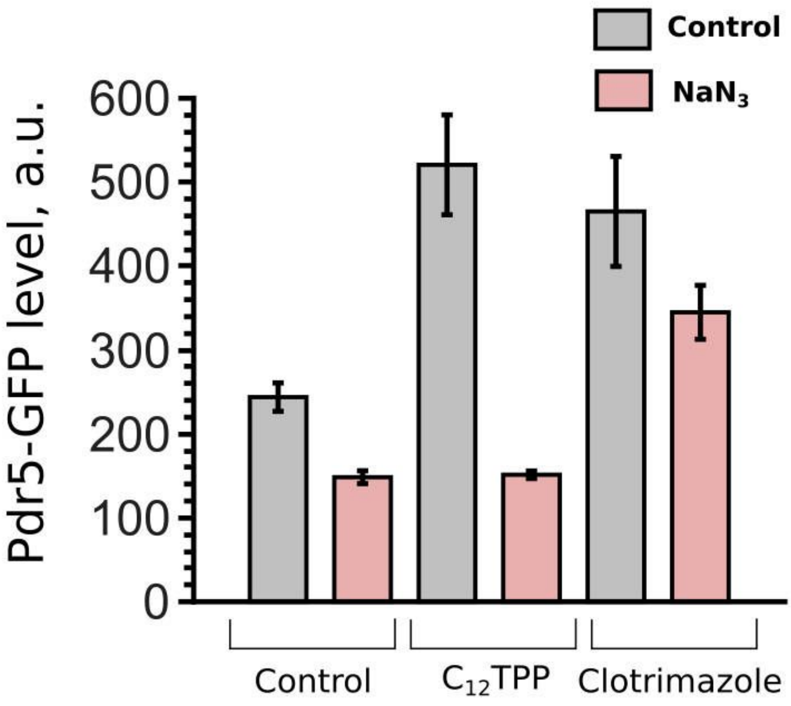

Figure S8. Sodium azide (NaN_3, 10 mM) inhibits Pdr5-GFP accumulation induced by C_{12}TPP (2.5 μM, incubation time 1 hour). Clotrimazole (20 μM) was used as a control to show that NaN_3 did not abolish protein synthesis. Mean ± SD is indicated (n = 3).

.
